# Cytochrome c Oxidase Subunit 5A (COX5A) Enhances Gastric Cancer Progression by Augmenting ATP Synthesis and Activating the PI3K/Akt Pathway

**DOI:** 10.1111/jcmm.70922

**Published:** 2025-11-03

**Authors:** Dongyan Li, Limin Zhou

**Affiliations:** ^1^ Human Resources Department First Affiliated Hospital of Jinzhou Medical University Jinzhou China; ^2^ Department of Infectious Diseases First Affiliated Hospital of Jinzhou Medical University Jinzhou China

**Keywords:** ATP synthesis, COX5A, gastric cancer, mitochondrial function, PI3K/Akt pathway

## Abstract

Gastric cancer (GC) is a lethal malignancy characterised by poor prognosis. In this study, we identify cytochrome c oxidase subunit 5A (COX5A) as a key metabolic driver and prognostic biomarker in GC. *COX5A* was upregulated in tumours and correlated with poor survival. Mechanistically, *COX5A* enhanced mitochondrial oxidative phosphorylation to elevate ATP production, activating PI3K/Akt signalling to drive proliferation, migration, and invasion. These effects were reversed by PI3K/Akt inhibitors. JC‐1 assays revealed *COX5A*‐mediated mitochondrial membrane potential elevation, indicating amplified bioenergetic output. In vivo, *COX5A* silencing suppressed xenograft tumour growth. Our results demonstrate *COX5A* orchestrates metabolic reprogramming and PI3K/Akt‐mediated progression in GC, positioning it as both a prognostic indicator and therapeutic target.

## 1 Introduction

Gastric cancer (GC) is one of the most prevalent and aggressive malignancies globally, accounting for a significant proportion of cancer‐related deaths [1, 2]. Despite advances in diagnostic and therapeutic strategies, the prognosis for GC remains poor, primarily due to its high metastatic potential and resistance to treatment [3, 4]. A hallmark of cancer progression is metabolic reprogramming, a process through which tumour cells alter their metabolism to meet the increased energy demands associated with rapid proliferation and survival in hostile microenvironments [5, 6].

Mitochondrial dysfunction and altered cellular metabolism have emerged as central drivers of oncogenesis, influencing critical processes including tumour growth, migration, and chemoresistance [7, 8]. One key player in mitochondrial function is cytochrome c oxidase (COX), the terminal enzyme in the mitochondrial electron transport chain (ETC), which is responsible for ATP generation via oxidative phosphorylation (OXPHOS) [9]. COX is a multi‐subunit complex composed of several catalytic subunits, with cytochrome c oxidase subunit 5A (COX5A) playing a crucial role in regulating mitochondrial ATP production and cellular energy homeostasis [10]. Recent studies suggest that alterations in *COX5A* expression and activity may contribute to the dysregulated energy metabolism observed in cancer cells [11], although its specific role in GC progression remains unclear.

In this study, we investigate the potential of *COX5A* as a key regulator of metabolic reprogramming in GC cells and its contribution to tumour progression. We hypothesize that *COX5A* facilitates GC progression by enhancing mitochondrial ATP synthesis through OXPHOS, thus supporting the bioenergetic demands of rapidly proliferating tumour cells. Additionally, we explore the interplay between *COX5A* and the phosphoinositide 3‐kinase (PI3K)/Akt signalling pathway, a critical regulator of cell survival, growth, and metabolism [12, 13]. Dysregulation of the PI3K/Akt pathway is frequently observed in various cancers, including GC, and is known to promote aggressive tumour behaviours, such as proliferation, invasion, and resistance to apoptosis [14, 15]. We propose that *COX5A* may influence the PI3K/Akt pathway, thereby linking mitochondrial function to key oncogenic signalling cascades involved in GC progression.

Recent studies have demonstrated that cancer cells often exhibit metabolic reprogramming, including increased reliance on mitochondrial oxidative phosphorylation for ATP generation, even in the presence of sufficient oxygen—a phenomenon known as the Warburg effect [16]. However, in many cancers, mitochondrial ATP synthesis via OXPHOS remains essential for supporting aggressive tumour growth and metastasis, suggesting that targeted modulation of mitochondrial function could offer new therapeutic opportunities. Given this, *COX5A* may emerge as a potential biomarker and therapeutic target for GC, as its upregulation could drive metabolic adaptations that enhance cancer cell survival and tumour progression.

In this manuscript, we present evidence supporting the role of *COX5A* in GC progression through its regulation of mitochondrial ATP synthesis and the PI3K/Akt signalling pathway. By analysing *COX5A* expression in GC tissues and cell lines, we correlate its expression with clinical outcomes in GC patients. We also conduct in vitro experiments, including gene silencing and *COX5A* overexpression, to explore its functional role in GC cell metabolism and tumourigenesis. Finally, we examine the potential of *COX5A* as a novel therapeutic target in GC, with implications for the development of strategies aimed at disrupting the metabolic dependencies of GC cells.

## 2 Methods

### 2.1 Data Acquisition and Processing

Transcriptomic data of gastric cancer (GC) and corresponding clinical data were obtained from The Cancer Genome Atlas (TCGA) database (https://portal.gdc.cancer.gov/). The dataset includes data from 34 adjacent normal tissue samples and 415 GC samples.

### 2.2 Differential Expression of COX5A in Gastric Cancer and Its Correlation With Clinical and Pathological Features

The expression of *COX5A* in various malignancies was evaluated using the TIMER 2.0 database (http://timer.cistrome.org/). *COX5A* mRNA expression in GC was analysed using data from the TCGA database. Matched tumour‐normal pairs were excluded from this analysis due to incomplete data. Additionally, the UALCAN database (http://ualcan.path.uab.edu/analysis.html) was used to assess the mRNA expression levels of *COX5A* in GC and explore its association with tumour stage, gender, age, pathological subtype, and regional lymph node involvement.

### 2.3 Analysis of COX5A Expression and Survival Prognosis of GC Patients

GC clinical data from TCGA were analysed using the survminer package in R software to plot Kaplan–Meier curves for overall survival (OS) analysis. Patients were stratified into high‐ and low‐expression groups based on the median *COX5A* mRNA expression level (high: *n* = 207, low: *n* = 208). To further investigate the clinical significance of *COX5A* in GC, we conducted a comprehensive analysis using TCGA data. Specifically, we evaluated the association between *COX5A* expression and patient survival by performing Cox proportional hazards regression (HR) analysis. This analysis incorporated various clinical factors, including age, pathological stage, and pathological grade. The survival data for GC patients from TCGA were stratified based on *COX5A* expression levels, and hazard ratios (HRs) were calculated to assess the impact of *COX5A* expression on overall survival.

### 2.4 Correlation Analysis of COX5A Expression and Clinicopathological Characteristics of GC Patients

RNA sequencing data from 413 GC tumour tissues and 36 adjacent normal tissues, along with comprehensive patient information (including age, sex, survival time, death status, TNM stage, etc.), was collected from the TCGA database. Expression analyses of *COX5A* mRNA in these datasets were conducted using the ggplot2 and ggpubr packages in R software (version 4.4.1). Additionally, the clinical correlation between *COX5A* expression levels and the pathological data of GC patients was examined with respect to gender, age, clinical stage, and N stage. For categorical variables with multiple groups (e.g., clinical stage, *N* stage), the Kruskal–Wallis test was employed. To assess the correlation between continuous *COX5A* expression and ordinal pathological stage, Spearman's rank correlation analysis was performed, and the correlation coefficient (*ρ*) along with its *p*‐value is reported.

### 2.5 Cell Lines

A human normal gastric mucosal cell line (GES‐1) and six human gastric cancer (GC) cell lines were sourced from different repositories. Specifically, GSU, SNU‐601, and IM‐95 were obtained from Shanghai Hongshun Biological Technology Co. Ltd. (Shanghai, China), a commercial distributor for international cell repositories. In contrast, GSE‐1, SNU‐1, and the remaining cell lines were acquired from the Cell Bank of the Chinese Academy of Sciences (Shanghai, China). All cell lines were cultured according to their specific growth requirements: SNU‐1 and SNU‐601 were maintained in suspension culture, while GSE‐1, GSU, and IM‐95 were cultured as adherent monolayers. All cells were grown in RPMI‐1640 medium supplemented with 10% fetal bovine serum (FBS), 100 U/mL penicillin, and 100 μg/mL streptomycin at 37°C in a 5% CO_2_ atmosphere. Suspension cultures were passaged by centrifugation, and adherent cultures were passaged via trypsinization.

### 2.6 Experimental Specimens

A total of 95 gastric cancer specimens, including cancerous tissues and adjacent non‐cancerous tissues (more than 5 cm away from the tumour margin), were collected from patients undergoing surgery at the First Affiliated Hospital of Jinzhou Medical University between January 2022 and December 2023. Data on gender, age, tumour diameter, number of lesions, presence of extramural invasion, lymph node metastasis, and TNM staging were also recorded. Among the patients, 85 had adenocarcinoma and 10 had non‐adenocarcinoma. All patients provided written informed consent prior to surgery.

### 2.7 Chemical Reagents and Antibodies

Luminescent ATP Detection Assay (ab113849), JC1 Mitochondrial Membrane Potential Assay Kit (ab113850), and Anti‐*COX5A* antibody (ab181226, 1:1000) were purchased from Abcam (Berlin, Germany). Phospho‐PI3K (Tyr458/Tyr199) (#17366, 1:1000), phospho‐Akt (Ser473) (#4060, 1:1000), and β‐Actin (#4967, 1:1000) antibodies were obtained from Cell Signalling Technology (Beverly, MA, USA). Ly294002 (purity 99.86%), a highly selective PI3K inhibitor, was obtained from Selleck Chemicals (Houston, TX, USA). Transwell culture chambers and Matrigel were purchased from Corning (Corning, NY, USA). TRIzol reagent was obtained from Thermo Fisher (Waltham, MA, USA).

### 2.8 RNA Extraction and Quantitative Real‐Time PCR (RT‐qPCR)

Total cellular RNA was extracted using TRIzol reagent according to the manufacturer's instructions. SYBR Green‐based RT‐qPCR was performed to assess *COX5A* mRNA expression. GAPDH was selected as the endogenous control for RT‐qPCR based on its well‐established stability for mRNA normalisation in gastric cancer cell lines and tissues in our experimental conditions. The following primer sequences were used: COX5A forward (5′‐AGGAGGAGGAGGAGGAGGA‐3′), COX5A reverse (5′‐CTTCTTCTTCTTCTTCTTC‐3′), GAPDH forward (5′‐GAAGGTGAAGGTCGGAGTC‐3′), and GAPDH reverse (5′‐GAAGATGGTGATGGGATTTC‐3′) (Integrated DNA Technologies, Coralville, IA, USA).

### 2.9 Western Blotting

Cellular proteins were extracted using radioimmunoprecipitation assay (RIPA) buffer supplemented with phenylmethylsulfonyl fluoride (PMSF). Protein concentration was quantified using a BCA protein assay kit. Proteins were separated by electrophoresis on a 10% SDS‐PAGE gel and transferred onto a PVDF membrane. The membrane was blocked with 5% bovine serum albumin (BSA) in TBST (TBS + Tween 20) and incubated overnight at 4°C with the primary antibody. For protein loading control, *β*‐Actin was used due to its consistent expression across our gastric cancer cell line models. After incubation, the membrane was treated with a goat anti‐rabbit horseradish peroxidase (HRP)‐conjugated secondary antibody at room temperature for 90 min. Following membrane washing, the proteins were detected using an ECL chemiluminescent reagent and developed in the dark. The relative expression levels of the target protein were analysed using ImageJ software. All experiments were repeated at least three times.

### 2.10 Immunohistochemical Streptavidin–Avidin Biotin Complex(SABC) Method for Detection of COX5A Protein Expression

Immunohistochemical staining was performed on 95 gastric cancer (GC) specimens and adjacent non‐cancerous tissues, as well as xenograft tumour tissues (for COX5A and p‐Akt analysis). The tissues were fixed in 10% formalin solution, followed by routine dehydration, clearing, and paraffin embedding. Sections were cut at a thickness of 4 μm, and antigen retrieval was performed using microwave treatment. The tissues were incubated with 3% hydrogen peroxide for quenching, followed by serum blocking. The primary antibody (against target proteins: *COX5A* or p‐Akt (Ser473)) was applied and incubated overnight at 4°C, followed by the addition of the secondary antibody. After DAB staining, the slides were counterstained with haematoxylin, and routine dehydration, clearing, and mounting with neutral resin were performed.

For quantitative analysis of *COX5A* and p‐Akt immunostaining, a standardised IHC scoring system (H‐score) was applied by two independent pathologists blinded to experimental groups. Five representative high‐power fields (HPFs, 400×) per sample were evaluated, focusing exclusively on viable tumour cells while excluding necrotic areas, tissue edges, and stromal components. Staining intensity was graded as: 0 (no staining), 1+ (weak, light yellow), 2+ (moderate, yellow‐brown), and 3+ (strong, brown); the percentage of positive tumour cells in 10% increments was recorded for each intensity. The H‐score was calculated per HPF using the formula: (1 × % 1+ cells) + (2 × % 2+ cells) + (3 × % 3+ cells), yielding a range of 0–300. The final *H*‐score per sample was the average of five HPF scores. To ensure rigour, 20% of samples were independently scored by both pathologists to confirm inter‐observer concordance (> 90%), with discrepancies (> 50‐point difference) resolved by joint re‐evaluation; remaining samples were divided for scoring. Final *H*‐scores were used for all statistical comparisons of protein expression.

### 2.11 Stable Cell Line Generation

Lentiviral vectors expressing COX5A‐targeting shRNA (shCOX5A‐1: 5′‐GCACTACCTGAAGGTCAAGAA‐3′; shCOX5A‐2: 5′‐GCTGGTCAAGATCATCCTCTA‐3′) or COX5A cDNA (GenBank: NM_004255.4) were constructed using pLKO.1‐puro and pLVX‐EF1α backbones, respectively. Lentiviruses were packaged in 293T cells by co‐transfection with psPAX2 and pMD2.G plasmids using Lipofectamine 3000 (Invitrogen, Carlsbad, CA, USA). Viral supernatants were collected at 48 and 72 h post‐transfection, concentrated by ultracentrifugation (50,000× *g*, 4°C, 2 h), and resuspended in PBS.

IM95 and SNU‐601 cells (high endogenous *COX5A* expression) were transduced with shCOX5A‐1 or shCOX5A‐2 lentivirus (MOI = 10) for 24 h, followed by puromycin selection (2 μg/mL, 14 days). AGS cells (low endogenous *COX5A* expression) were transduced with *COX5A*‐overexpressing lentivirus and selected via FACS sorting for mCherry^+^ cells (pLVX‐EF1α‐COX5A‐IRES‐mCherry). Control cells were transduced with non‐targeting shRNA (shCtrl) or empty vector (Vector). Knockdown and overexpression efficiency were validated by qRT‐PCR and Western blotting prior to in vivo studies.

### 2.12 ATP Synthesis Assay

ATP synthesis was measured using a luminescence‐based ATP detection kit. Briefly, GC cells with *COX5A* knockdown or overexpression were seeded at a density of 1 × 10^5^ cells per well in 24‐well plates and cultured for 24 h. After treatment, the cells were lysed using 100 μL of cell lysis buffer per well, and the lysates were incubated at room temperature for 10 min. ATP levels were then quantified using the luminescence reagent according to the manufacturer's instructions. Luminescence was measured using a microplate reader at an emission wavelength of 510 nm. The results were normalised to the total protein concentration, which was determined using the BCA protein assay. All experiments were performed in triplicate.

### 2.13 JC‐1 Mitochondrial Membrane Potential Assay

Mitochondrial membrane potential was assessed using the JC‐1 staining kit (Thermo Fisher Scientific). GC cells with *COX5A* knockdown or overexpression were seeded at a density of 1 × 10^5^ cells per well in 6‐well plates and incubated for 24 h. After the indicated treatment, cells were incubated with 1 μM JC‐1 dye for 30 min at 37°C in a CO₂ incubator. Following incubation, cells were washed twice with PBS and analysed by flow cytometry (BD FACSCalibur). The fluorescence emission of JC‐1 was measured at 530 nm (green, indicating depolarized mitochondria) and 590 nm (red, indicating polarised mitochondria). The ratio of red/green fluorescence intensity was calculated to assess the mitochondrial membrane potential. Cells treated with FCCP (Carbonyl cyanide‐4‐(trifluoromethoxy)phenylhydrazone) were used as a positive control for mitochondrial depolarization.

### 2.14 Immunofluorescence

After treatment with specific inducers or inhibitors to modulate the expression levels of the target proteins, cultured GC cells were fixed with paraformaldehyde and permeabilized with Triton X‐100 for antibody staining. The cells were then blocked with BSA to prevent non‐specific binding and incubated overnight with primary antibodies against *COX5A*, p‐PI3K, and p‐AKT. After washing, the cells were incubated with suitable fluorescently labelled secondary antibodies, and cell nuclei were stained with DAPI. Samples were imaged using fluorescence microscopy. Fluorescence intensity was quantified using image analysis software.

### 2.15 Cell Proliferation Assay

Cell proliferation was assessed using the MTT assay. Briefly, 0.8 × 10^4^ cells were plated in 96‐well plates and incubated for different time periods. MTT reagent was then added, and absorbance was measured at 494 nm using a microplate reader.

### 2.16 Colony Formation Assay

A total of 0.7 × 10^3^ cells were plated into individual wells of 6‐well plates and cultured in complete medium for 14 days. The cells were then fixed with 1% methanol and stained with 1% crystal violet. Colonies with a diameter ≥ 3 mm were visualised using a digital camera and quantified.

### 2.17 Cell Migration and Invasion Assays

The migration and invasion capabilities of GC cells were assessed using Transwell chambers, which were coated with Matrigel for the invasion assay. A total of 0.5 × 10^5^ cells, resuspended in serum‐free medium, were added to the upper chambers, while the lower chambers were filled with complete medium. After incubation for 24 h, the cells remaining on the upper surface of the membrane were removed, while those adhered to the lower surface were fixed, stained, photographed, and counted.

### 2.18 Xenotransplantation Studies

Stably transduced cells (1 × 10^7^ cells in 100 μL DMEM) were subcutaneously injected into the dorsal flanks of 6‐week‐old female nude mice (nu/nu). The mice were randomly divided into four experimental groups (*n* = 5 per group): (1) shNC group: IM95/SNU‐601 cells expressing non‐targeting shRNA as a negative control; (2) shCOX5A group: IM95/SNU‐601 cells with *COX5A* knockdown, generated by pooling two independent validated shRNAs (shCOX5A‐1 and shCOX5A‐2) at a 1:1 ratio to ensure consistent knockdown efficiency and minimise off‐target effects; (3) oe‐NC group: AGS cells transfected with empty vector; and (4) oe‐COX5A group: AGS cells overexpressing *COX5A*.

Tumour growth was monitored over 28 days, with tumour volume measured every 3 days using digital callipers and calculated using the formula: volume = 0.5 × length × width^2^. At the study endpoint, all mice were humanely euthanized by cervical dislocation under isoflurane anaesthesia (5% for induction and 2% for maintenance). Following euthanasia, tumours were carefully excised, weighed, and immediately snap‐frozen in liquid nitrogen before storage at −80°C for subsequent biochemical analyses.

Prior to in vivo experiments, the knockdown efficiency of both shCOX5A‐1 and shCOX5A‐2 was rigorously validated through quantitative PCR (qPCR) and western blot analysis. The pooled shRNA approach was employed to enhance the reliability of the knockdown phenotype and reduce potential off‐target effects, as recommended for RNA interference studies. This validation ensured that the observed phenotypic changes could be confidently attributed to *COX5A* knockdown rather than experimental artefacts.

### 2.19 Statistical Analysis

All experiments were performed in triplicate and repeated at least three times, unless otherwise stated. Statistical analyses were conducted using R software (v4.4.1; https://www.r‐project.org/) with survminer (v0.4.9), ggplot2 (v3.4.4), and ggpubr (v0.6.0) for survival plots and visualisation; GraphPad Prism (v6.0; https://www.graphpad.com/) for statistical tests; and ImageJ (v1.53 t; https://imagej.nih.gov/ij/) for Western blot and immunohistochemical quantification. Data are expressed as mean ± SD/SEM, with significance (*p* < 0.05) determined by ANOVA and chi‐square tests. For correlation analyses involving continuous and ordinal variables, Spearman's rank correlation was used, and the correlation coefficient (*ρ*) is reported along with the corresponding *p*‐value. The original data underlying all graphs and representative images (including uncropped Western blot membranes) are available from the corresponding author upon reasonable request.

## 3 Results

### 3.1 COX5A Gene in Gastric Cancer and Its Differential Analysis and Prognosis

**FIGURE 1:**
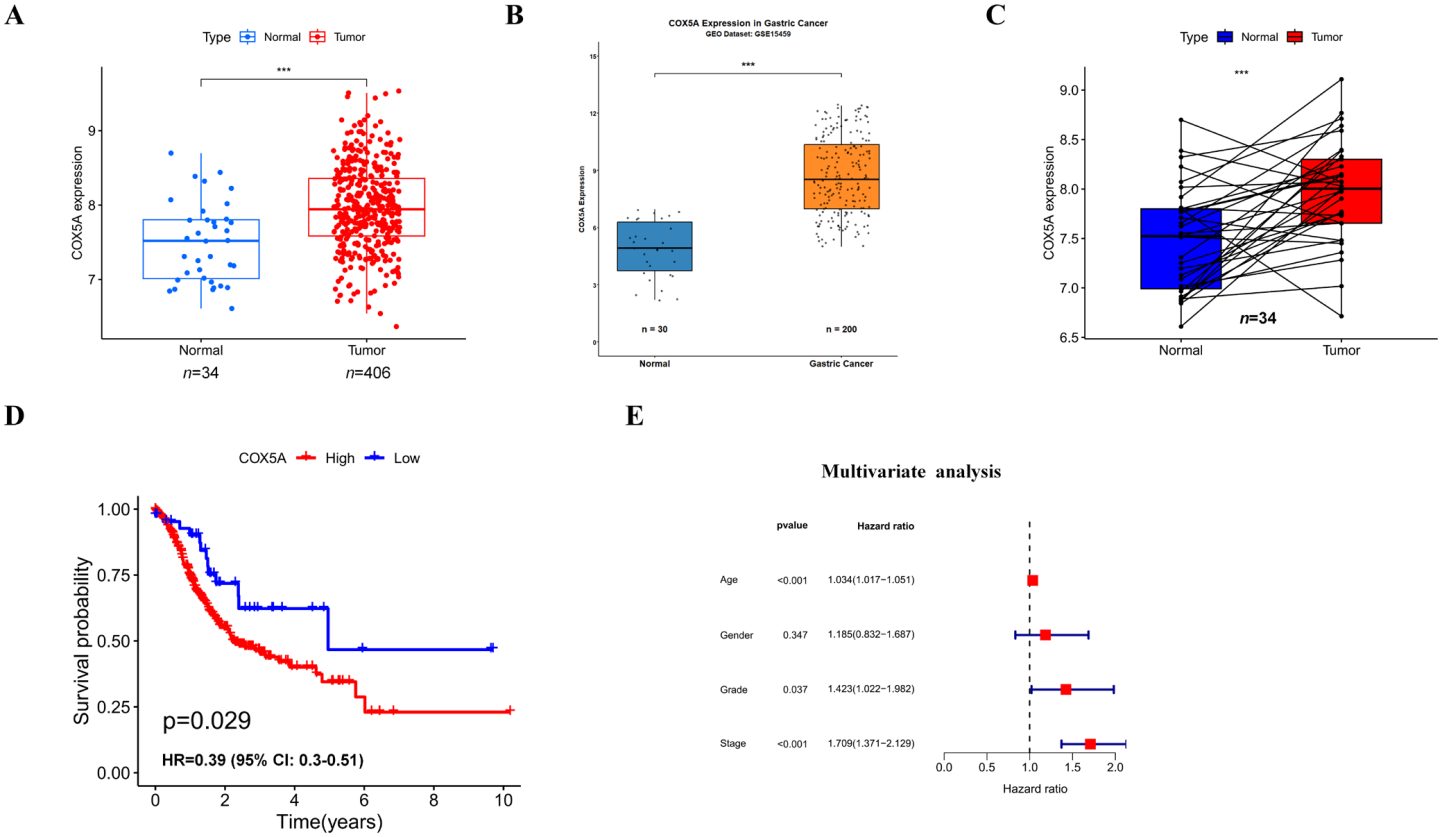
*COX5A* Expression and Its Prognostic Implications in Gastric Cancer (STAD). (A) Wilcoxon rank‐sum test analysis of *COX5A* expression in STAD tumour tissues (*n* = 406) versus adjacent normal tissues (*n* = 34). *COX5A* expression was significantly higher in tumour tissues (*p* < 0.001). (B) Validation of COX5A mRNA upregulation in an independent GEO cohort (GSE15459), showing expression in GC tissues (*n* = 200) versus non‐tumour tissues (*n* = 30). ***p < 0.001. (C) Paired analysis of COX5A mRNA expression in matched tumour and adjacent normal tissues from the same patients (*n* = 34 pairs). ***p < 0.001. (D) Kaplan–Meier survival curves for overall survival (OS) in GC patients stratified by high (*n* = 207) versus low (*n* = 208) COX5A mRNA expression. The hazard ratio (HR) and 95% confidence interval (CI) are displayed on the graph. *p < 0.05. (E) Forest plot of multivariate Cox regression analysis for overall survival, identifying COX5A expression as an independent prognostic factor after adjustment for age, stage, and grade. Data Presentation and Statistical Analysis: Data in panels A–C are presented as box plots (median, quartiles, and range). Statistical significance was determined by the Student's *t*‐test (A, B) and paired *t*‐test (C). Survival analysis (D) was performed using the log‐rank test. A *p*‐value of less than 0.05 was considered statistically significant. The specific sample size (*n*) for each analysis is indicated within the respective figure panel.

Using the UALCAN database, a total of 449 samples were retrieved with ‘STAD’ as the search term. The analysis revealed that the expression level of *COX5A* mRNA in STAD tissue (*n* = 415) was significantly higher than that in adjacent normal tissues (*n* = 34, *p* < 0.001, Figure 1A). Furthermore, the Wilcoxon rank‐sum test showed that the expression of *COX5A* in STAD tissues (*n* = 406) was significantly elevated compared to adjacent normal tissues (*n* = 34, *p* < 0.001, Figure 1B). Additionally, a significant difference in *COX5A* expression was observed between tumour tissues and normal tissues within the same samples (*p* < 0.001, Figure 1C).

Kaplan–Meier survival analysis was performed to assess overall survival (OS) in gastric cancer patients. The results demonstrated a significant association between *COX5A* expression and OS. Specifically, patients with high *COX5A* expression exhibited significantly shorter overall survival compared to those with low *COX5A* expression (Figure 1D). Multivariate Cox regression analysis (adjusted for age, stage, and grade) identified *COX5A* as an independent prognostic factor for overall survival in gastric cancer (HR = 1.52, *p* < 0.01) (Figure 1E).

### 3.2 Association of COX5A Expression With Clinicopathological Features in Gastric Cancer

**FIGURE 2:**
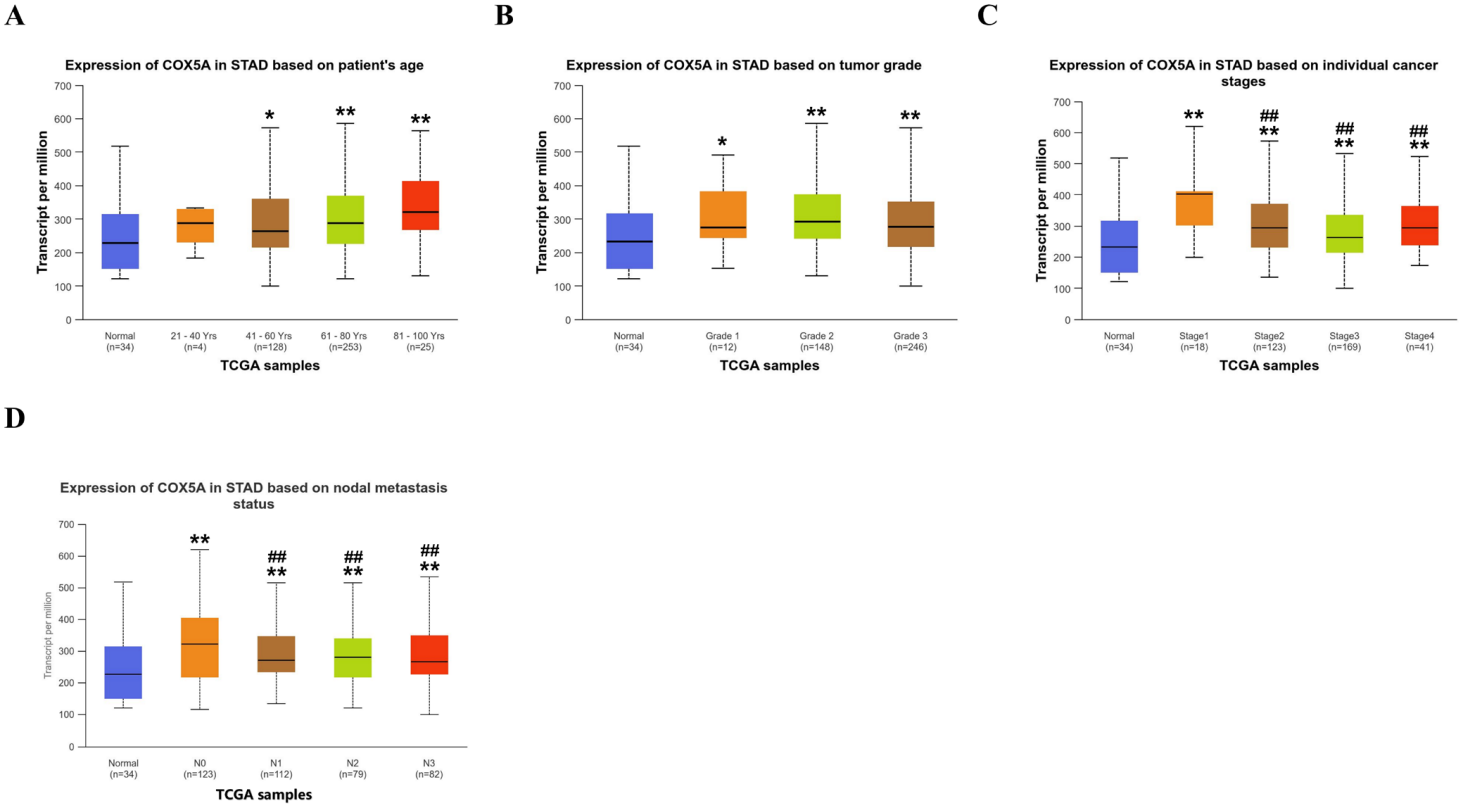
Association of *COX5A* Expression with Clinicopathological Features in Gastric Cancer. (A) Relationship between COX5A expression and patient age, showing no significant association. (B–D) COX5A expression levels across different pathological categories in gastric cancer tissues compared to normal adjacent tissues: (B) Tumour grade (G1–G3), (C) Clinical stage (Stage I–III), (D) Lymph node metastasis status (N stage). Data are presented as mean ± SD. Sample sizes: normal tissues, *n* = 34; tumour tissues, *n* = 406 (TCGA‐STAD dataset). Statistical analysis was performed using the Kruskal–Wallis test with Dunn's post hoc test for multiple comparisons. Significance notations:*p < 0.05, **p < 0.01, compared to the Normal group (B–D); ^#^ *p* < 0.05, ^##^ *p* < 0.01 compared to the Stage I group (C) or the N0 group (D). A non‐monotonic trend was observed in clinical stages (C), with the highest expression in Stage I.

Analysis of public genomic data from the TCGA stomach adenocarcinoma dataset revealed a distinct pattern of *COX5A* expression during gastric cancer progression. *COX5A* expression was significantly elevated in tumour tissues compared to normal tissues. Furthermore, its expression levels varied significantly across different tumour grades, clinical stages, and N stages (Figure 2B–D). Notably, *COX5A* expression was highest in Stage I tumours and subsequently decreased in Stages II and III (Figure 2C), suggesting a potential role in early tumourigenesis. No clear association was observed with patient age (Figure 2A). The relationship between *COX5A* expression and pathological stage was assessed using the Kruskal–Wallis test, which revealed a significant difference among the groups (*p* < 0.001). Given the non‐monotonic trend observed (highest in Stage I), Spearman's rank correlation was deemed inappropriate for assessing a continuous relationship.

To validate the clinical relevance of *COX5A*, we analysed a cohort of 95 gastric cancer patients from our institution. We first confirmed that *COX5A* expression was significantly higher in tumour tissues compared to paired adjacent normal tissues (*p* < 0.001).

We next investigated whether high *COX5A* expression was linked to key clinicopathological parameters by comparing tumours with high versus low *COX5A* expression levels. Our analysis demonstrated that high *COX5A* expression was significantly linked to larger tumour size (*p* < 0.05), deeper invasion depth (*p* < 0.01), presence of lymph node metastasis (*p* < 0.01), and advanced TNM stage (*p* < 0.01, Table 1). No significant association was found with patient gender or age (*p* > 0.05, Table 1).

**TABLE 1:** Association between *COX5A* expression and clinicopathological characteristics in gastric cancer patients (*n* = 95).

Clinicopathological characteristic	Category	*n*	*COX5A* expression (Mean ± SD)	Statistical index	*p*
Gender	Male	58	1.35 ± 0.28	*t* = 0.89	0.374
	Female	37	1.29 ± 0.31		
Age (years)	< 60	46	1.32 ± 0.30	*t* = −0.45	0.653
	≥ 60	49	1.34 ± 0.29		
Tumor size (cm)	< 5	52	1.25 ± 0.24	*t* = −2.15	**0.034**
	≥ 5	43	1.41 ± 0.33		
Depth of invasion (T stage)	T1–T2	41	1.18 ± 0.22	*t* = −4.87	**< 0.001**
	T3–T4	54	1.44 ± 0.29		
Lymph node metastasis (N stage)	No (N0)	38	1.15 ± 0.20	*t* = −5.92	**< 0.001**
	Yes (N1–N3)	57	1.45 ± 0.28		
TNM stage	I–II	44	1.17 ± 0.21	*t* = −5.41	**< 0.001**
	III–IV	51	1.46 ± 0.27		

*Note:* Association between *COX5A* expression (treated as a continuous variable) and clinicopathological characteristics was analyzed using Student's *t*‐test. SD, standard deviation. Bolded *p*‐values indicate statistical significance (*p* < 0.05).

### 3.3 COX5A Is Overexpressed in Clinical GC Tissues and Cell Lines, Supporting Its Detectability by IHC

**FIGURE 3:**
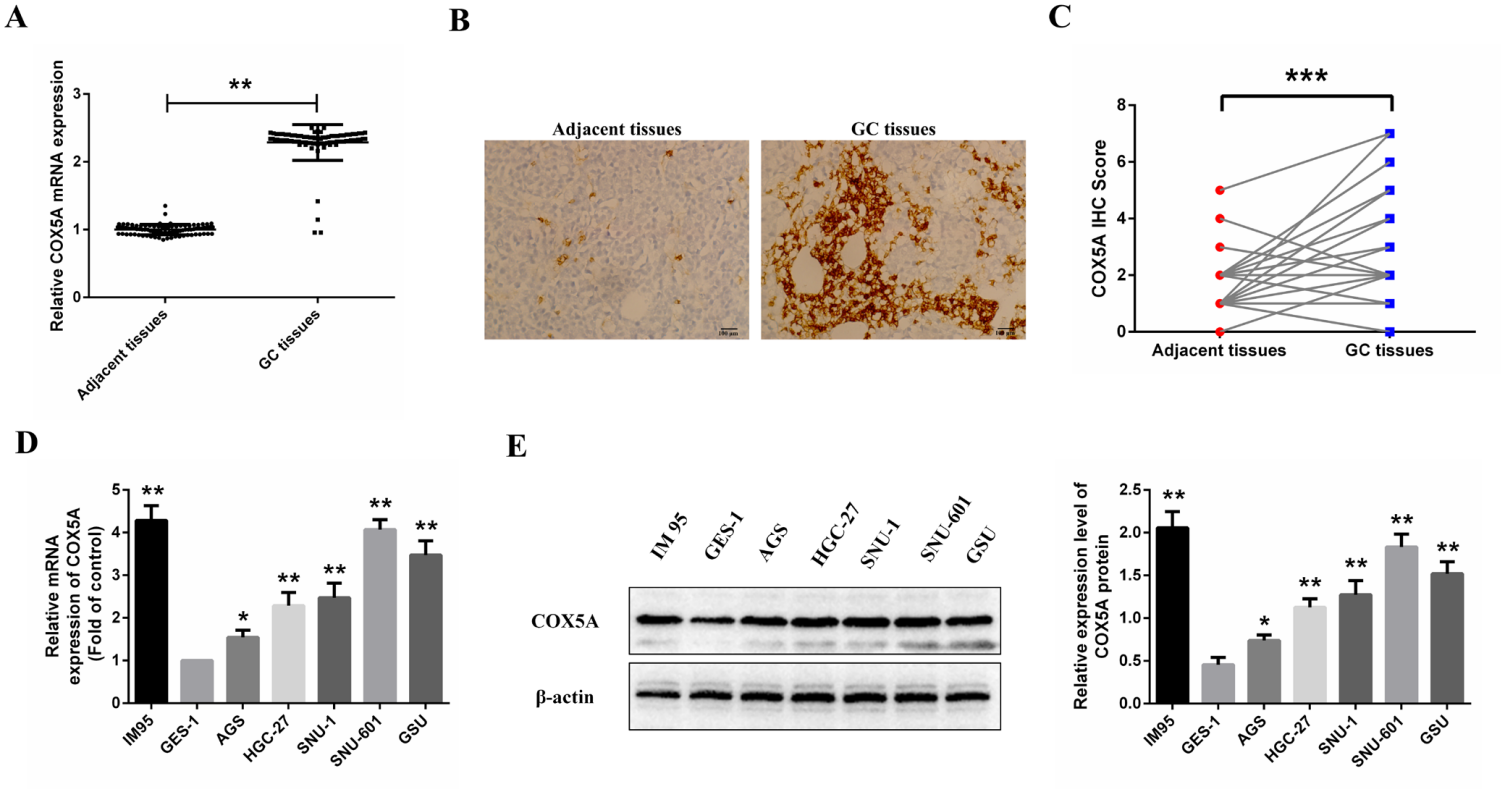
COX5A Expression in Gastric Cancer (GC) Tissues and Cell Lines. (A) Quantitative real‐time PCR (qRT‐PCR) analysis of COX5A mRNA expression in tumour tissues and adjacent normal tissues from 95 GC patients. COX5A mRNA expression was markedly elevated in tumour tissues compared to normal tissues (*p < 0.05). (B) Representative immunohistochemistry (IHC) images showing COX5A protein expression in GC tumour tissues and adjacent normal tissues. COX5A protein was predominantly localised in the cytoplasm. Scale bars: 100 μm. (C) Statistical analysis of COX5A protein expression in tumour and adjacent normal tissues. The positive expression rate of COX5A was markedly elevated in tumour tissues compared to normal tissues (**p < 0.01). (D) RT‐PCR analysis of COX5A mRNA expression in GC cell lines (HGC‐27, GSU, AGS, SNU‐1, SNU‐601, and IM95) and the normal gastric epithelial cell line GES‐1. COX5A mRNA expression was significantly elevated in GC cell lines compared to GES‐1, with AGS cells showing relatively lower expression and IM95 and SNU‐601 cells exhibiting higher expression. (E) Western blot analysis of COX5A protein expression. Black lines indicate where the blot was cropped for concise presentation; full‐length blots are provided in the Supporting Information. *p < 0.05 and **p < 0.01 for GC tissue vs. Adjacent tissue in panels A and C; *p < 0.05 and **p < 0.01 for comparisons with the GES‐1 cell line in panels D and E.

In this study, quantitative real‐time PCR (qRT‐PCR) was used to measure *COX5A* mRNA expression in tumour and adjacent normal tissues from 95 gastric cancer (GC) patients. Quantitative analysis demonstrated significantly elevated *COX5A* mRNA expression in tumour tissues relative to adjacent normal tissues (Figure 3A).

To further validate these findings, immunohistochemistry (IHC) was performed to evaluate *COX5A* protein expression in GC tissues. *COX5A* protein was predominantly localised in the cytoplasm. The positive expression rate of *COX5A* in tumour tissues was significantly higher (74.74%, 71/95) compared to adjacent normal tissues (12.63%, 12/95), with this difference being statistically significant (Figure 3B,C). Notably, the robust detection of *COX5A* protein in routine FFPE clinical specimens underscores its practicality as a prognostic biomarker assessable by standard immunohistochemistry in diagnostic biopsies.

We also collected cell samples and assessed *COX5A* mRNA expression by RT‐PCR, while *COX5A* protein expression was analysed using Western blotting. The results demonstrated that both *COX5A* mRNA and protein expression were significantly elevated in GC cell lines compared to the normal gastric epithelial cell line GES‐1 (Figure 3D,E). Among the GC cell lines (HGC‐27, GSU, AGS, SNU‐1, SNU‐601, and IM95), AGS cells exhibited relatively lower *COX5A* mRNA and protein expression, whereas IM95 and SNU‐601 cells displayed higher levels of *COX5A* mRNA and protein expression. Based on these observations, we selected AGS, IM95, and SNU‐601 cells for subsequent experiments.

### 3.4 COX5A Expression Modulates ATP Synthesis and Mitochondrial Membrane Potential in Gastric Cancer Cells

**FIGURE 4:**
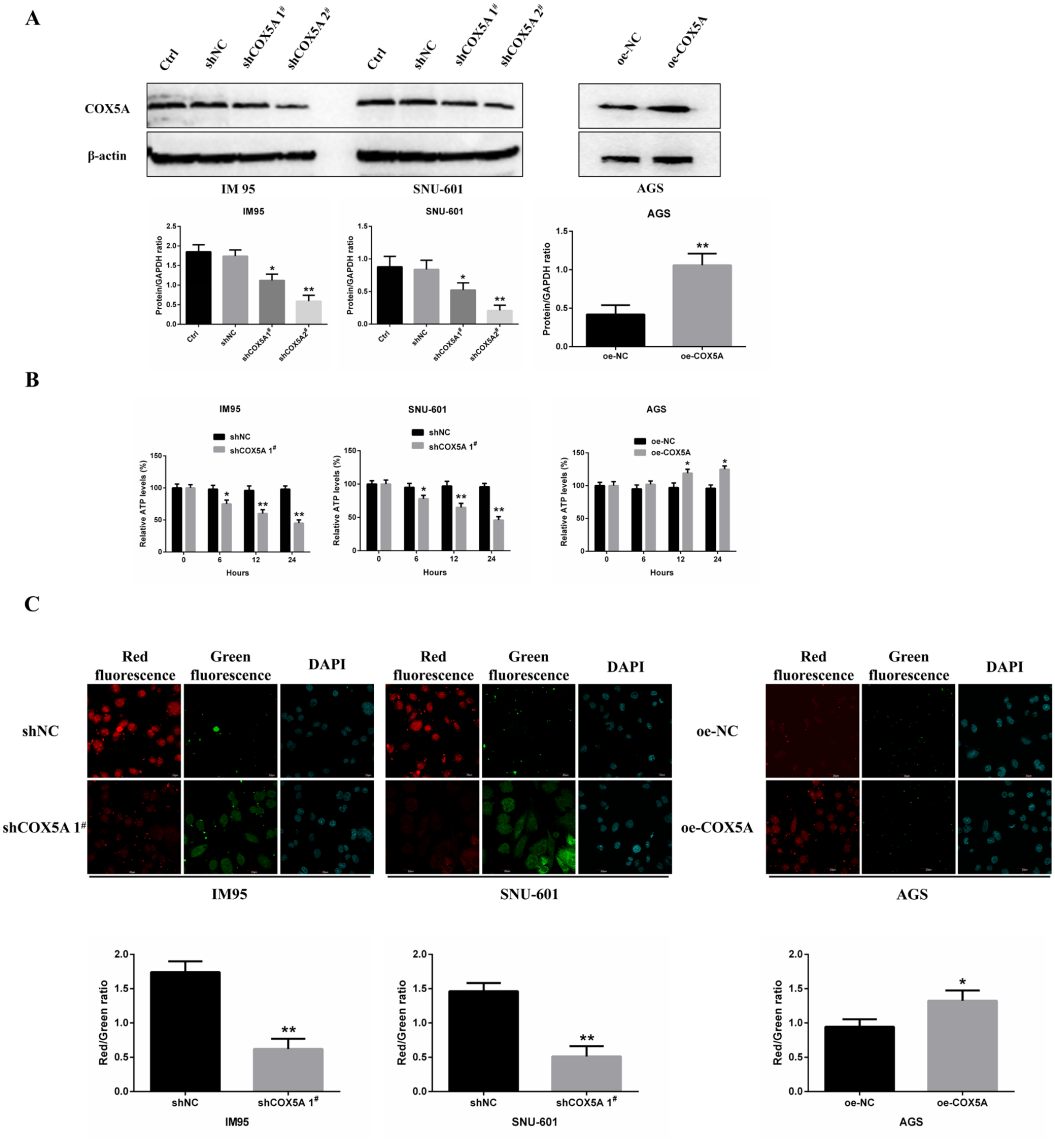
Role of *COX5A* in Oxidative Phosphorylation and ATP Synthesis in Gastric Cancer Cells. Expression of *COX5A* in GC cell lines. (A) Western blot analysis of COX5A protein expression following shRNA‐mediated knockdown in IM95 and SNU‐601GC cells (high COX5A expression) and overexpression in AGS cells (low COX5A expression). COX5A expression was significantly reduced in IM95 and SNU‐601GC cells and significantly increased in AGS cells, confirming efficient gene modulation. Uncropped images of all Western blots are available in the Supporting Information. (B) ATP synthesis levels measured at 0, 6, 12, and 24 h using an ATP Synthesis Assay. Downregulation of COX5A in IM95 and SNU‐601GC cells resulted in a significant decrease in ATP synthesis, with lower ATP levels observed at 12 and 24 h compared to the shNC group. Conversely, overexpression of COX5A in AGS cells led to a significant increase in ATP synthesis, with higher ATP levels at 12 and 24 h compared to the oe‐NC group. (C) Mitochondrial membrane potential assessed using the JC‐1 Mitochondrial Membrane Potential Assay. In IM95 and SNU‐601GC cells with COX5A downregulation, a decrease in red fluorescence intensity and an increase in green fluorescence intensity were observed, indicating reduced mitochondrial membrane potential. In AGS cells overexpressing COX5A, red fluorescence intensity was significantly increased, suggesting enhanced mitochondrial membrane potential compared to the oe‐NC group. Scale bars: 20 μm. Significance notation: *p < 0.05, **p < 0.01.

Given the well‐established primary role of *COX5A* in mitochondrial complex IV and oxidative phosphorylation (OXPHOS) [17, 18], we hypothesized that its overexpression in GC directly enhances mitochondrial bioenergetics. To investigate the role of *COX5A* in oxidative phosphorylation and ATP synthesis in gastric cancer cells, we first downregulated *COX5A* expression in IM95 and SNU‐601GC cells, which exhibit high levels of *COX5A* expression, using shRNA. *COX5A* was also overexpressed in AGS cells. Protein expression levels were assessed by Western blotting to evaluate the efficiency of gene knockdown and overexpression. The results showed a significant reduction in *COX5A* expression in IM95 and SNU‐601GC cells, while *COX5A* expression was significantly upregulated in AGS cells (Figure 4A).

To assess the functional consequences of *COX5A* modulation, ATP synthesis levels were measured at 0, 6, 12, and 24 h using an ATP Synthesis Assay. In IM95 and SNU‐601GC cells, downregulation of *COX5A* led to a significant decrease in ATP synthesis, starting at 6 h, with ATP levels at 12 and 24 h being significantly lower than those in the shNC group. In contrast, in AGS cells overexpressing *COX5A*, ATP synthesis began to increase at 6 h, and at 12 and 24 h, ATP levels were markedly higher than those in the oe‐NC group (Figure 4B).

To further validate the impact of *COX5A* on mitochondrial function, mitochondrial membrane potential was assessed using the JC‐1 Mitochondrial Membrane Potential Assay. In IM95 and SNU‐601GC cells with *COX5A* downregulation, a significant reduction in red fluorescence intensity and an increase in green fluorescence intensity of the JC‐1 dye were observed, indicating a decrease in mitochondrial membrane potential. Conversely, in AGS cells overexpressing *COX5A*, the red fluorescence intensity of JC‐1 was significantly increased compared to the oe‐NC group, suggesting an enhancement of mitochondrial membrane potential (Figure 4C).

### 3.5 COX5A Knockdown Suppresses GC Cell Proliferation, Migration, and Invasion

**FIGURE 5:**
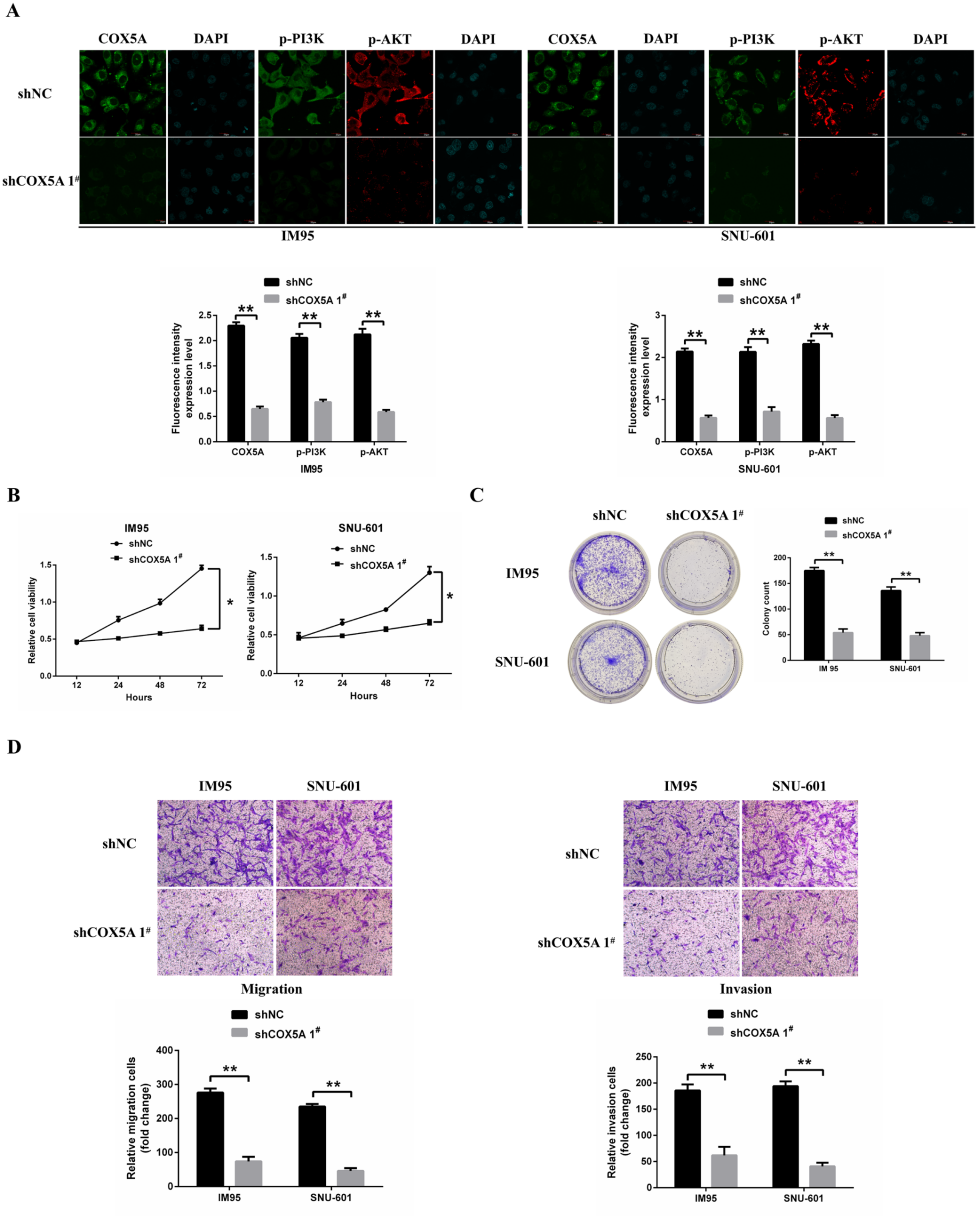
*COX5A* knockdown inhibits GC cell proliferation, migration, and invasion. (A) Immunofluorescence staining was performed to confirm successful shRNA‐mediated *COX5A* knockdown and evaluate its effect on PI3K and AKT phosphorylation in IM95 and SNU‐601 cells. Scale bars: 20 μm. (B, C) The effect of *COX5A* knockdown on GC cell growth was assessed using the MTT method (72 h) and colony formation assays (14 days). (D) Transwell assays were conducted to quantify migration and invasion capacities in *COX5A*‐knockdown cells and control (shNC) cells. Scale bars: 100 μm. Data are presented as mean ± SD (*n* = 3 replicates per group). *p < 0.05, **p < 0.01.

To elucidate the role of *COX5A* in the proliferation, migration, and invasion of gastric cancer (GC) cells, we employed shRNA to silence *COX5A* expression in IM95 and SNU‐601 GC cell lines, both of which exhibit high endogenous *COX5A* levels. The efficiency of *COX5A* knockdown was confirmed at the protein level through immunofluorescence analysis, which demonstrated a significant reduction in *COX5A* expression following shRNA‐mediated silencing. Additionally, we assessed the effect of *COX5A* knockdown on key signalling pathways and observed a notable decrease in the phosphorylation of both PI3K and AKT, indicating effective inhibition of these pathways (Figure 5A).

To assess the impact of *COX5A* silencing on cell proliferation, MTT assays and colony formation assays were performed to evaluate the proliferative capacity and tumourigenic potential of the cells. The results revealed that *COX5A* knockdown significantly inhibited both proliferation and clonogenic growth in IM95 and SNU‐601 cells (Figure 5B,C).

To further investigate whether downregulation of *COX5A* affects the metastatic potential of GC cells, Transwell assays were conducted. The results showed a significant reduction in migration and invasion capacities after *COX5A* knockdown, compared to the shNC‐transfected cells (Figure 5D).

### 3.6 Overexpression of COX5A Promotes Proliferation, Migration, and Invasion of GC Cells

**FIGURE 6:**
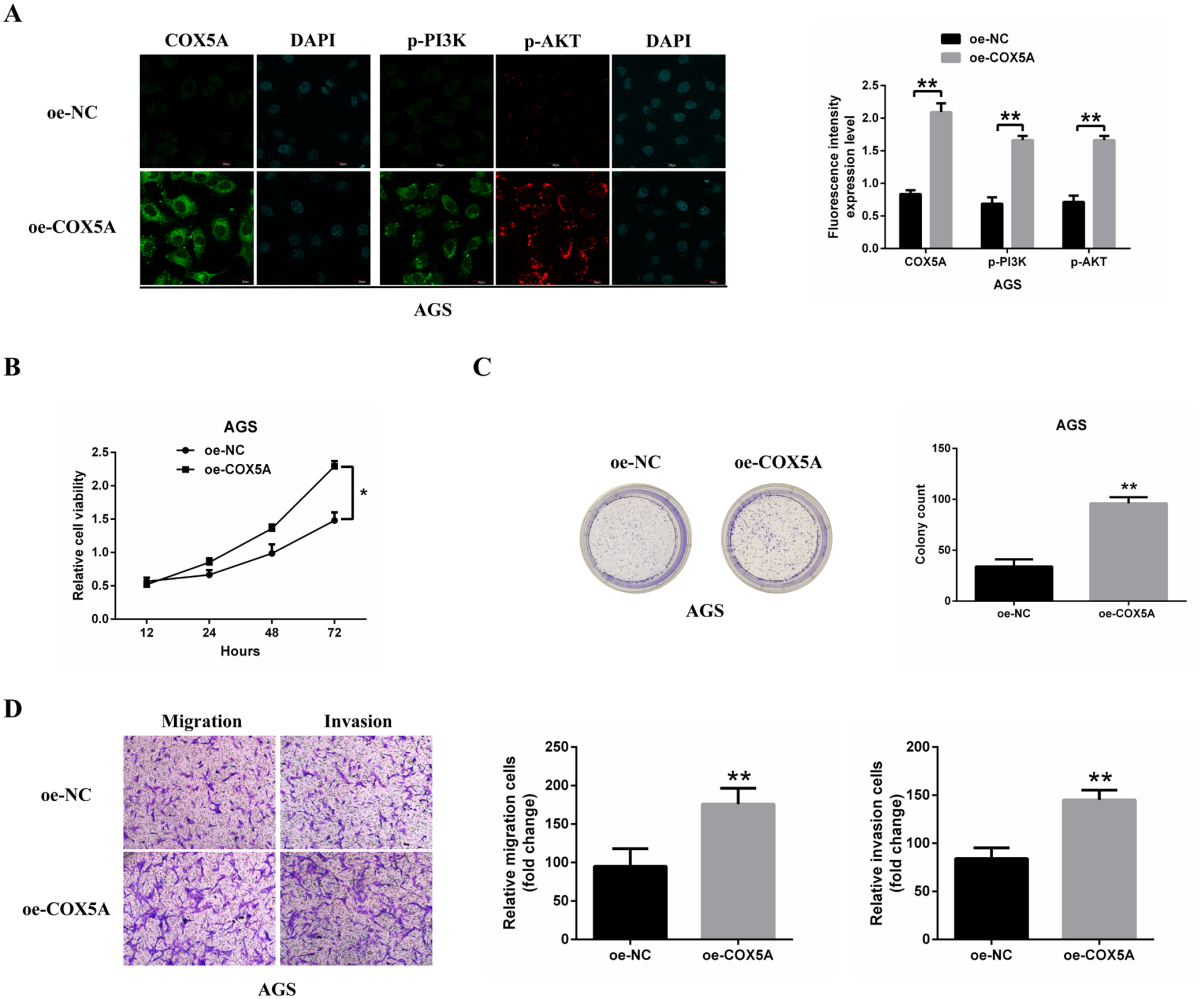
*COX5A* overexpression promotes GC cell proliferation, migration, and invasion. (A) Immunofluorescence staining was performed to confirm successful *COX5A* overexpression and evaluate its effect on PI3K and AKT phosphorylation in AGS cells. Scale bars: 20 μm. (B, C). Results of MTT assays (72 h) and colony formation assays (14 days) examining the proliferation kinetics and tumourigenic potential of *COX5A*‐overexpressing and control (oe‐NC) AGS cells. (D) Transwell assays were conducted to quantify the migration and invasion of *COX5A*‐overexpressing and control AGS cells. Scale bar: 100 μm. Data are expressed as mean ± SD (*n* = 3 replicates per group). *p < 0.05, **p < 0.01.

To further investigate the relationship between *COX5A* and GC cell proliferation, migration, and invasion, we overexpressed *COX5A* in AGS cells, which exhibit low levels of *COX5A* expression. Immunofluorescence analysis confirmed successful overexpression of *COX5A*, along with increased phosphorylation of PI3K and AKT compared to the control (oe‐NC) cells (Figure 6A).

MTT assay results showed that overexpression of *COX5A* significantly promoted AGS cell proliferation (Figure 6B,C). Additionally, Transwell assays revealed a marked increase in the number of migrating and invading cells in the *COX5A* overexpression group compared to the control group (Figure 6D).

### 3.7 COX5A Modulation in Stable Xenografts Promotes Tumour Growth

**FIGURE 7:**
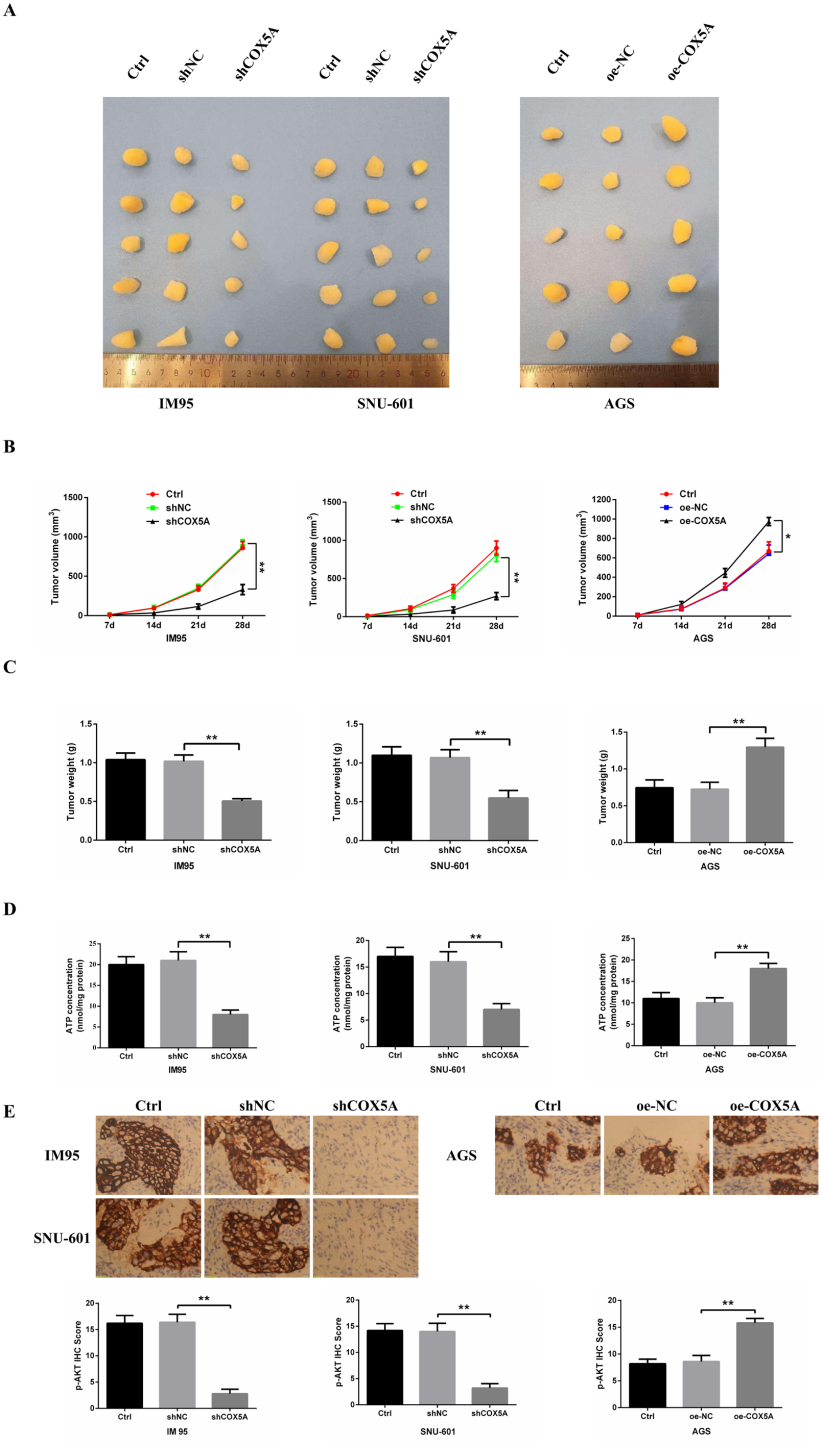
Effects of *COX5A* Modulation on Tumour Growth in Gastric Cancer Xenograft Models. (A) Representative images of xenograft tumours derived from IM95, SNU‐601, and AGS cells with modulated *COX5A* expression. Tumours from IM95 and SNU‐601 cells with *COX5A* downregulation were significantly smaller compared to the control group, while tumours from AGS cells with *COX5A* overexpression were markedly larger than the control group. (B) Tumour growth curves showing changes in tumour volume over time. Downregulation of *COX5A* in IM95 and SNU‐601 cells resulted in significantly slower tumour growth compared to the control group. In contrast, *COX5A* overexpression in AGS cells led to accelerated tumour growth. (C) Comparison of tumour weight at the endpoint of the experiment. Tumours from IM95 and SNU‐601 cells with *COX5A* downregulation exhibited significantly lower weights, while tumours from AGS cells with *COX5A* overexpression showed markedly elevated weights compared to their respective control groups. (D) ATP levels in tumour tissues. *COX5A* knockdown reduced ATP content compared to sh‐NC. *COX5A* overexpression increased ATP levels versus oe‐NC. (E) Immunohistochemical (IHC) analysis of p‐AKT (Ser473) expression in xenograft tumours. *COX5A* knockdown reduced p‐AKT staining intensity (brown signal), while *COX5A* overexpression enhanced p‐AKT activation. Scale bars: 20 μm. Significance notation: *p < 0.05, **p < 0.01.

Xenograft models were established using lentivirally transduced GC cells with stable *COX5A* modulation. Tumours derived from shCOX5A‐expressing IM95/SNU‐601 cells exhibited significantly reduced volume and weight versus shCtrl (Figure 7A–C). Conversely, COX5A‐OE tumours from AGS cells showed marked increases in volume and weight compared to Vector controls.

ATP levels were significantly decreased in shCOX5A tumours but elevated in oe‐COX5A tumours (Figure 7D). Quantitative IHC revealed reduced p‐AKT (Ser473) in shCOX5A tumours and enhanced activation in COX5A‐OE tumours (Figure 7E), directly linking *COX5A* to PI3K/AKT pathway activation.

### 3.8 COX5A Enhances ATP Synthesis and Mitochondrial Function via PI3K/AKT Activation to Promote Gastric Cancer Progression

**FIGURE 8:**
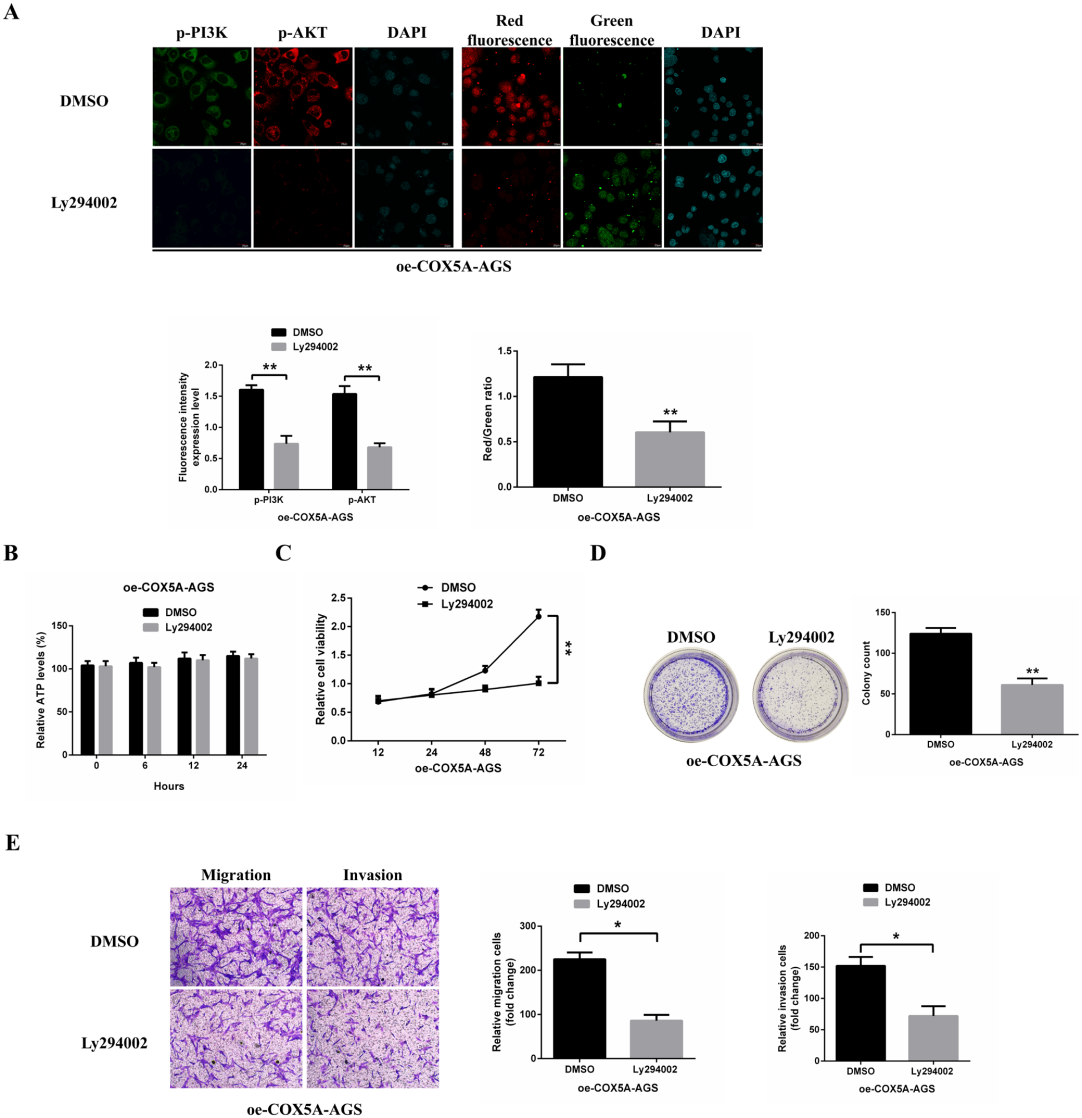
*COX5A* Promotes Gastric Cancer Progression Through PI3K/AKT‐Dependent Mitochondrial Regulation. (A) Immunofluorescence analysis of PI3K/AKT phosphorylation status in AGS cells treated with PI3K inhibitor Ly294002 (20 μM, 24 h). Ly294002 effectively abolished PI3K (p85α) and AKT (Ser473) phosphorylation, as evidenced by diminished fluorescence intensity (Scale bar: 20 μm). JC‐1 staining revealed mitochondrial depolarization in Ly294002‐treated cells, characterised by decreased red/green fluorescence ratio (*p* < 0.01). (B) ATP quantification assay demonstrated sustained ATP levels in Ly294002‐treated cells despite PI3K/AKT inhibition. (C) MTT assay showed Ly294002 treatment (20 μM) completely abrogated the proliferative advantage conferred by *COX5A* overexpression (*p* < 0.001) at 72 h post‐treatment. (D) Colony formation assay showing reduced clonogenic capacity in *COX5A*‐overexpressing cells treated with Ly294002 (*p* < 0.01). (E) Transwell assays (8 μm pore) revealed Ly294002 abolished *COX5A*‐enhanced migratory and invasive capacities (*p* < 0.001 for both), stained with 0.1% crystal violet. Significance notation: *p < 0.05, **p < 0.01.

Our findings indicate that *COX5A* activates the PI3K/AKT signalling pathway, which subsequently promotes proliferation, migration, and invasion in gastric cancer (GC) cells. To confirm these findings, we treated AGS cells with Ly294002, a potent PI3K inhibitor. Immunofluorescence analysis revealed that Ly294002 effectively suppressed the phosphorylation of PI3K and AKT. Furthermore, JC‐1 staining analysis showed that Ly294002 treatment significantly reduced the mitochondrial membrane potential, indicating a disruption in mitochondrial function (Figure 8A). However, despite the inhibition of PI3K/AKT signalling by Ly294002, ATP synthesis remained unaffected, further supporting the hypothesis that *COX5A* promotes ATP synthesis via a PI3K/AKT‐dependent mechanism to drive gastric cancer progression (Figure 8B). MTT and colony formation assays further demonstrated that in the presence of Ly294002, *COX5A* overexpression failed to induce cell proliferation (Figure 8C,D). Similarly, Transwell assays showed that *COX5A* overexpression did not enhance migration or invasion in cells treated with Ly294002 (Figure 8E).

## 4 Discussion

Our study establishes *COX5A* as a dual‐functional regulator of gastric cancer (GC) progression, serving as a critical nexus between mitochondrial bioenergetics and oncogenic signalling pathways. The integration of clinical bioinformatics with functional validation reveals *COX5A*'s central role in disease aggressiveness and its potential utility as both a prognostic biomarker and therapeutic target.

The overexpression of *COX5A* in GC tissues, coupled with its strong correlation to diminished overall survival across multiple cohorts, positions this mitochondrial complex IV subunit as a clinically relevant prognostic indicator. Critically, our IHC analysis confirms that *COX5A* protein is readily detectable in archival clinical samples, positioning it as a viable candidate for integration into future prognostic models based on biopsy material. This aligns with its established role in maintaining cytochrome c oxidase activity and oxidative phosphorylation [17, 18], processes critical for sustaining the metabolic plasticity of cancer cells. Our findings extend current understanding by demonstrating that *COX5A*‐driven mitochondrial bioenergetics not only fulfil the heightened energy demands of proliferating tumour cells but also create a permissive metabolic environment for GC progression. Such metabolic reprogramming mirrors the adaptive strategies employed by cancer cells to thrive in nutrient‐scarce microenvironments [19, 20], with *COX5A* emerging as a gatekeeper of mitochondrial efficiency in malignant transformation. Notably, the enhanced mitochondrial ATP production mediated by *COX5A* aligns with broader observations linking mitochondrial hyperactivity to cancer cell survival, proliferation, and resistance to apoptosis—a hallmark of aggressive malignancies [21, 22, 23, 24].

Beyond its canonical role in energy metabolism, our mechanistic investigations uncover *COX5A*'s capacity to directly engage with the PI3K/Akt signalling axis—a pathway frequently hijacked in GC pathogenesis [25, 26, 27]. This dual functionality distinguishes *COX5A* from other mitochondrial complex subunits, as its signalling activity operates independently of respiratory chain functions. The observed dissociation between *COX5A*‐mediated metabolic support and PI3K/Akt activation challenges the conventional view of mitochondrial proteins as mere metabolic regulators, instead positioning *COX5A* as a bifunctional molecular switch. This paradigm is reinforced by pharmacological inhibition studies showing pathway‐selective effects, where PI3K/Akt blockade does not compromise mitochondrial ATP synthesis. Such functional independence suggests that *COX5A* may coordinate complementary pro‐tumourigenic processes through distinct molecular interfaces, a phenomenon increasingly recognised in mitochondrial‐nuclear crosstalk [28].

The translational implications of these findings are twofold. Clinically, *COX5A*'s prognostic power across GC subtypes and its conserved association with poor outcomes in other malignancies [29] advocate for its integration into multi‐parameter prognostic models. Therapeutically, *COX5A* represents a unique vulnerability node, as its dual roles in metabolism and signalling provide opportunities for combinatorial targeting strategies. While current PI3K/Akt inhibitors face challenges with compensatory metabolic adaptation, simultaneous disruption of *COX5A*'s bioenergetic and signalling functions could circumvent such resistance mechanisms. This approach aligns with emerging paradigms in precision oncology that emphasise dual‐pathway inhibition to counteract tumour adaptability [30, 31].

Beyond its canonical role in energy metabolism, our mechanistic investigations uncover *COX5A*'s capacity to directly or indirectly engage with the PI3K/Akt signalling axis… The precise molecular link between *COX5A* and PI3K/Akt activation remains to be fully elucidated. We speculate that increased ATP production may modulate the AMPK/mTOR axis or directly influence Akt phosphorylation through energy‐sensing mechanisms [32]. Furthermore, while we focused on the PI3K/Akt pathway, *COX5A*‐mediated alterations in mitochondrial function, such as reactive oxygen species (ROS) generation [33] or modulation of calcium signalling, could potentially influence other oncogenic pathways like HIF‐1α or NF‐κB [34], representing fruitful areas for future research. While this study establishes the baseline oncogenic role of *COX5A* in GC, an important future direction will be to investigate the dynamics of its expression during therapeutic interventions. Given that metabolic reprogramming is a hallmark of cancer progression and therapy resistance [35], it is plausible that *COX5A*‐driven mitochondrial energetics contributes to acquired resistance in GC. This is particularly relevant as the PI3K/AKT/mTOR pathway—a key regulator of cell survival frequently dysregulated in GC—is known to interact with metabolic processes and confer resistance to therapies [36]. Monitoring *COX5A* expression levels in serial biopsies could therefore reveal its potential as a predictive biomarker for treatment response, a critical need in the personalised management of GC [37, 38].

This work repositions *COX5A* from a routine metabolic component to a multifunctional GC driver with biomarker and therapeutic potential. The mechanistic uncoupling of its metabolic and signalling roles provides a framework for developing context‐specific interventions. Future studies should address *COX5A* isoform specificity, evaluate its role in therapeutic resistance, and explore its interaction with tumour microenvironmental factors. A key limitation of the current study is the lack of in vivo validation using PI3K inhibitors in *COX5A*‐overexpressing xenograft models. Such experiments would provide critical support for the proposed mechanism and are a priority for our subsequent investigations. Validation in prospective clinical cohorts will be essential to translate these findings into actionable diagnostic and therapeutic strategies.

## Author Contributions

L. Z. contributed to the conceptualization and methodology, while D. L. handled the writing, review, and editing of the manuscript.

## Ethics Statement

This study was approved by the Medical Research Ethics Committee of the First Affiliated Hospital of Jinzhou Medical University (Approval No. 202355). Informed consent was obtained from all participants in accordance with ethical guidelines. All experimental procedures were carried out in compliance with the relevant regulations and guidelines, including those outlined in the Declaration of Helsinki. Xenotransplantation experiments were conducted in adherence to the ARRIVE guidelines 2.0 (2020) and were approved by the Experimental Animal Ethics Committee of Jinzhou Medical University (Protocol No. 20231236). All animal procedures conformed to the principles set forth in the NIH Guide for the Care and Use of Laboratory Animals (8th edition, 2011).

## Consent

The authors have nothing to report.

## Conflicts of Interest

The authors declare no conflicts of interest.

## Supporting information


**Figure S1:** jcmm70922‐sup‐0001‐FigureS1.png.

**Data S1:** jcmm70922‐sup‐0002‐DataS1.zip.

**Data S2:** jcmm70922‐sup‐0003‐DataS2.docx.

## Data Availability

The RNA sequencing data analyzed in this study were derived from the TCGA‐STAD project in the Genomic Data Commons (GDC) portal (https://portal.gdc.cancer.gov/). All data generated in this study are included in this published article and its Supporting Information files. The original datasets supporting the findings are available from the corresponding author upon reasonable request.
