# Supplementary figures and images for: Cytochrome c Oxidase Subunit 5A (COX5A) Enhances Gastric Cancer Progression by Augmenting ATP Synthesis and Activating the PI3K/Akt Pathway
Source: J Cell Mol Med. 2025 Nov 3;29(21):e70922. doi: 10.1111/jcmm.70922 (PMC12582873; doi:10.1111/jcmm.70922)

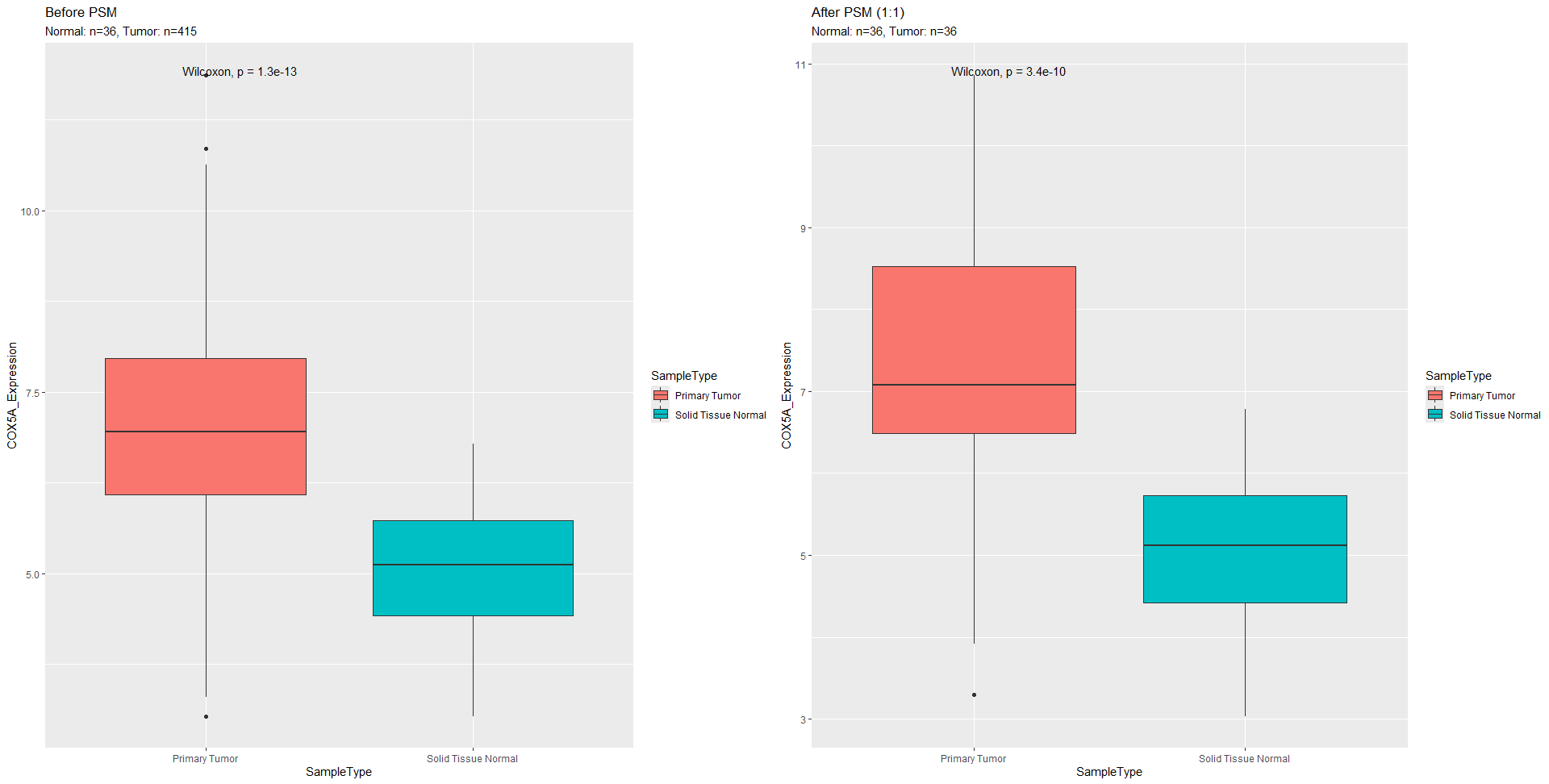

Supplement: Supplementary file 1 — Figure S1: jcmm70922‐sup‐0001‐FigureS1.png. [file JCMM-29-e70922-s003.png]
