# Supplementary material for: Cytochrome c Oxidase Subunit 5A (COX5A) Enhances Gastric Cancer Progression by Augmenting ATP Synthesis and Activating the PI3K/Akt Pathway
Source: J Cell Mol Med. 2025 Nov 3;29(21):e70922. doi: 10.1111/jcmm.70922 (PMC12582873; doi:10.1111/jcmm.70922)

**Supplementary Figure Legends**

**Fig. S1**. Full-length Western blot images of COX5A and GAPDH corresponding to cropped regions in Figure 3E.


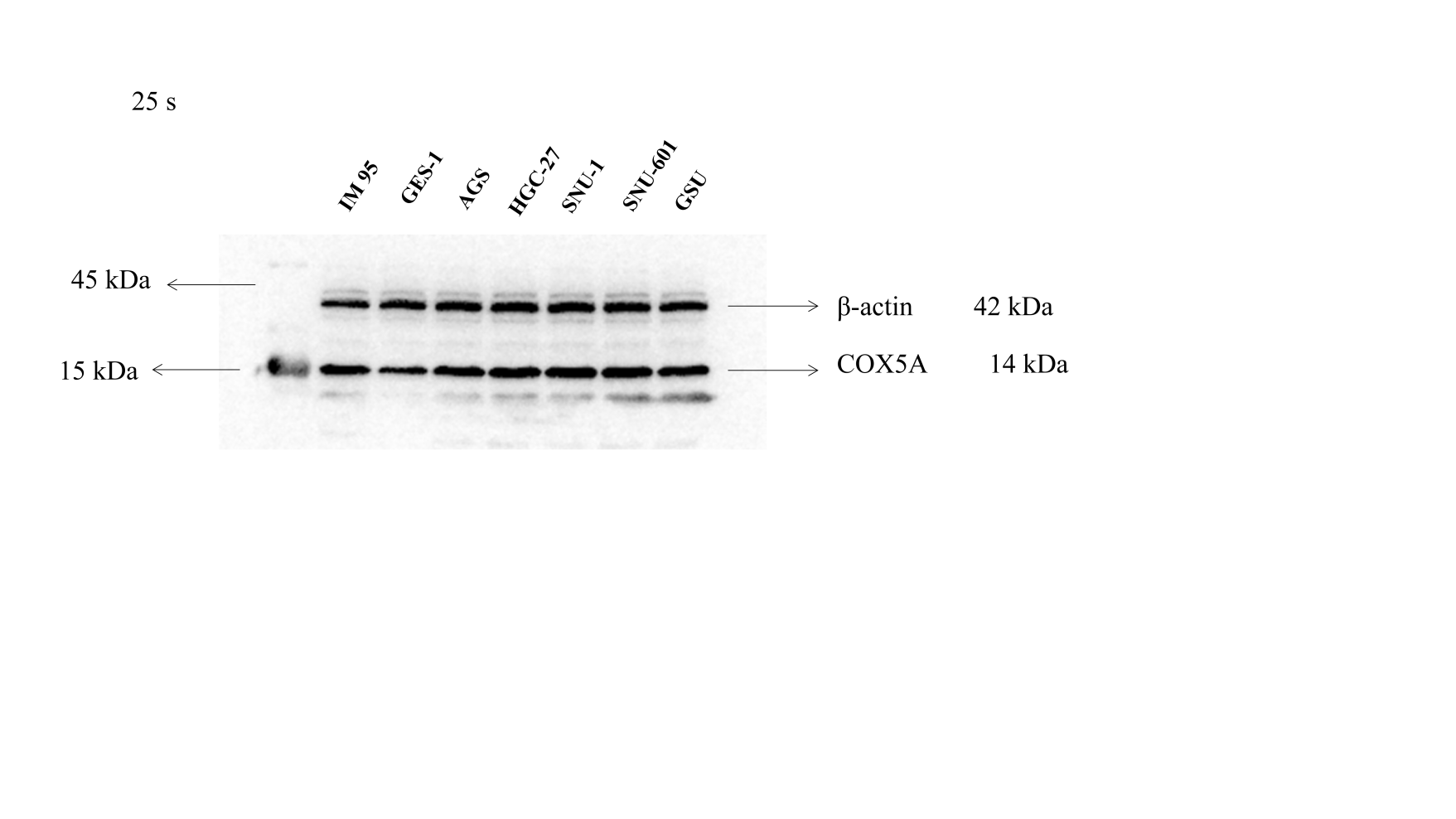


**Fig. S2**. Full-length Western blot images of COX5A and GAPDH corresponding to cropped regions in Figure 4A.


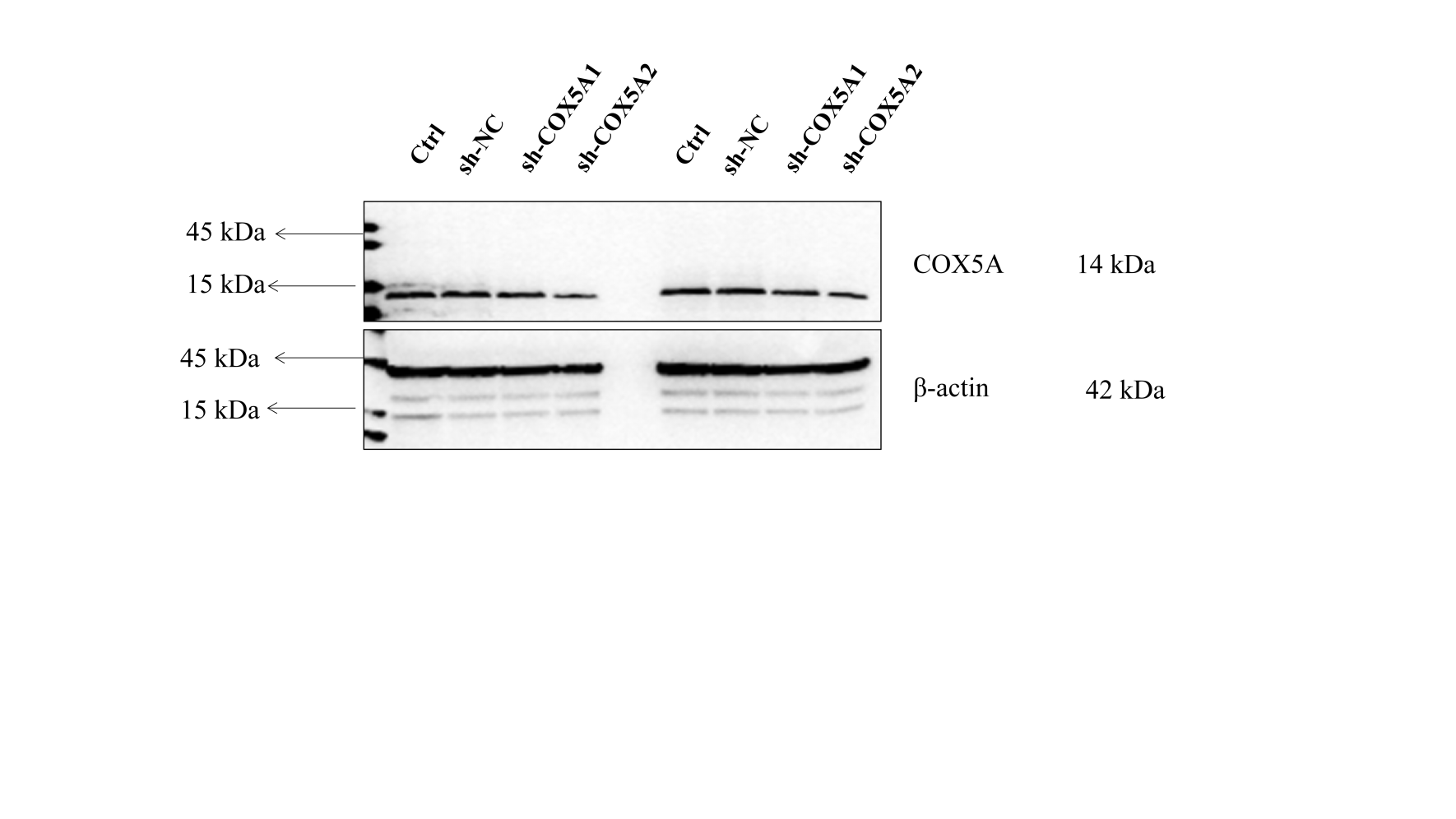

Supplement: Supplementary file 3 — Data S2: jcmm70922‐sup‐0003‐DataS2.docx. [file JCMM-29-e70922-s001.docx]
